# Coronary calcium score improves the estimation for pretest probability of obstructive coronary artery disease and avoids unnecessary testing in individuals at low extreme of traditional risk factor burden: validation and comparison of CONFIRM score and genders extended model

**DOI:** 10.1186/s12872-018-0912-3

**Published:** 2018-08-29

**Authors:** Minghui Wang, Yujie Liu, Xiujun Zhou, Jia Zhou, Hong Zhang, Ying Zhang

**Affiliations:** 1 Department of Cardiology, Tianjin Chest Hospital, 261 Taierzhuangnan Road, Tianjin, 300000 China; 2 Department of Radiology, Tianjin Chest Hospital, Tianjin, China; 3 Institute of Cardiovascular Diseases, Tianjin Chest Hospital, Tianjin, China

**Keywords:** Coronary computed tomographic angiography, Pretest probability of obstructive coronary artery disease, Low extreme of risk factor, Coronary calcium score, Unnecessary testing

## Abstract

**Background:**

Reliability of models for estimating pretest probability (PTP) of obstructive coronary artery disease (CAD) has not been investigated in individuals at low extreme of traditional risk factor (RF) burden. Thus, we sought to validate and compare CONFIRM score and Genders extended model (GEM) among these individuals.

**Methods:**

We identified symptomatic individuals with 0 or 1 RF who underwent coronary calcium scan and coronary computed tomographic angiography (CCTA). Follow-up clinical data were also recorded. PTP of obstructive CAD for every individual was estimated according to CONFIRM score and GEM, respectively. Area under the receiver operating characteristic curve (AUC), integrated discrimination improvement (IDI), net reclassification improvement (NRI) and Hosmer–Lemeshow (H-L) test were used to assess the performance of models.

**Results:**

There were 1201 individuals with 0 RF and 2415 with 1 RF. The AUC for GEM was significantly larger than that for CONFIRM score, no matter in individuals with 0 (0.843 v.s. 0.762, *p* < 0.0001) or 1 (0.823 v.s. 0.752, *p* < 0.0001) RF. Compared to CONFIRM score, GEM demonstrated positive IDI (5% in individuals with 0 RF and 8% in individuals with 1 RF), positive NRI (41.50% in individuals with 0 RF and 40.19% in individuals with 1 RF), better prediction of clinical events and less discrepancy between observed and predicted probabilities, resulting in a significant decrease of unnecessary testing, especially in negative individuals.

**Conclusion:**

In individuals at low extreme of traditional RF burden of CAD, the addition of coronary calcium score provided a more accurate estimation for PTP and application of GEM instead of CONFIRM score could avoid unnecessary testing.

## Background

Recent studies have indicated that clinical value of a test for the diagnosis of obstructive coronary artery disease (CAD) depended on the pretest probability (PTP) [1–3]. Considering this, current guidelines regard the estimation of PTP as an initial and important step in the evaluation of a symptomatic individual with suspected CAD [4, 5]. Updated Diamond-Forrester method (UDFM), a traditional age, sex and chest pain typicality-based approach to the PTP of obstructive CAD on invasive coronary angiography [6], is currently recommended by the European Society of Cardiology (ESC) [4]. However, several studies determined that UDFM seemed to overestimate the PTP of obstructive CAD, especially in low risk populations [7–9].

With modern statistical methods and multicenter data from populations who underwent coronary computed tomographic angiography (CCTA), new models, e.g. CONFIRM score [10] and Genders extended model (GEM) [11], were developed and what’s more, the addition of coronary calcium score (CCS) in GEM dramatically improved the estimation of PTP [7, 8]. However, neither CONFIRM score nor GEM has been systematacially validated in symptomatic individuals at low extreme of traditional risk factor (RF) burden, for whom the selection of an appropriate diagnostic strategy is important but difficult [12].

Thus, we aim to validate and compare the two proposed models and investigated whether or not the addition of CCS would avoid unnecessary testing among symptomatic individuals with 0 or 1 RF from a cohort of Chinese patients who underwent CCTA.

## Methods

### Study population

Full details for the study cohort have been published previously [7]. This is a retrospective and observational cohort of 5743 patients who underwent CCTA for stable chest pain. Individuals without acute coronary syndrome, previous CAD or coronary revascularization, unassessable segments due to motion artifact, atrial fibrillation, aortic disease, New York Heart Association class III or IV heart failure, age > 90 years old, pacemaker lead or missing data were enrolled between December 2014 and December 2016. This subgroup analysis among individuals with 0 or 1 RF was approved by the ethics committees of the local institutions and informed consent was obtained from all individual participants included in the study.

### Data collection and definitions

As part of the baseline examination, we collected information about traditional RFs, including smoking, hypertension, diabetes, and hyperlipidemia. Hypertension was defined as blood pressure of ≥140/90 mmHg or requiring antihypertensive treatment. Hyperlipidemia was defined as total cholesterol of ≥220 mg/dL, low-density lipoprotein cholesterol of ≥140 mg/dL, fasting triglycerides of ≥150 mm/dL or the need for antihyperlipidemic agents. Diabetes was defined as fasting glucose levels over 7 mmol/L or current treatment with either diet, oral glucose lowering agents, or insulin. Smoking was defined as current smoking or smoking in past 6 months. Family history of CAD was defined as myocardial infarction or cardiac death in a first-degree relative.

Chest pain was classified as typical angina if the following criteria were present: substernal chest pain, provoked by physical exertion or emotion, and relieved by rest or nitroglycerin. Atypical angina was defined by 2 of those criteria, and nonanginal chest pain if only 0 or 1 of 3 were present [13].

We validated and compared 2 regression models as previously developed and reported. CONFIRM score included age, sex, type of chest pain, diabetes, hypertension, family history of CAD and smoking [10]. GEM included age, sex, type of chest pain, dyslipidaemia, diabetes, hypertension, smoking and CCS [11]. We chose the low prevalence setting model when using GEM.

### CCTA and CCS

Details of CCS and CCTA scan have been previously described [7]. CCS was determined using the Agatston method [14]. In CCTA image analyses, all segments ≥2 mm in diameter were identified and analyzed using the CAD-RADS(TM) Coronary Artery Disease - Reporting and Data System [15]. Obstructive CAD was defined as present if a patient had at least one lesion with ≥50% diameter stenosis or any non-assessable segments due to severe calcification.

### Clinical outcomes

Follow-up information was obtained by phone call and/or physician visit after CCTA. The major adverse cardiovascular event (MACE) was composed of cardiac death, nonfatal myocardial infarction (MI), unstable angina hospitalization and late revascularization. All events were adjudicated via review of hospital records independently by 2 cardiologists who were blinded to the results of baseline testing in consensus. Cardiac death was defined as any death caused by cardiac disease or for which no other cause could be found. MI was defined when at least 2 of the following 3 criteria were met: chest pain or equivalent symptom complex, positive cardiac biomarkers, or typical ECG changes [16]. Late revascularizations (> 60 days after CCTA) are more likely to be associated with CAD progression.

### Statistical analysis

Individuals were categorized as having 0 or 1 of the following traditional RF: smoking, diabetes, hypertension and hyperlipidemia. Student’s t tests or Mann Whitney U tests (for continuous variables) and Chi-square tests or Fisher’s exact tests (for count variables) were used to compare baseline characteristics. To validate and compare CONFIRM score and GEM, the ability of discrimination, classification and calibration are essential in the present study. Discrimination is the degree to which a model separates between positive and negative individuals and we calculated the area under receiver-operator characteristic curve (AUC) [17] and integrated discrimination improvement (IDI) [18]. Classification evaluates whether a model correctly classifies positive individuals into higher categories of PTP and negative ones into lower categories. Based on a reclassification table using PTP categories < 15%, 15–85%, and > 85% [4], the net reclassification improvement (NRI) [18] was assessed. Calibration measures agreement of observed and predicted probability. Hosmer–Lemeshow (H-L) tests divided patients into ten groups according to deciles of PTP, then a chi-square statistic (H-L χ^2^) was calculated to evaluate how well model fit the obstructive CAD observed by CCTA [19]. All statistical analysis was performed by MedCalc (version 15.2.2; MedCalc Software, Mariakerke, Belgium) and SAS (version 9.2; SAS Institute Inc., Cary, North Carolina). Two-tailed *p* < 0.05 was considered statistically significant.

## Results

Table 1 shows the baseline characteristics of the study cohort by RF burden and presence of obstructive CAD on CCTA. There were 1201 individuals with 0 RF, of whom 363 (30%) were found to have obstructive CAD on CCTA. The mean age was 56.26 years and 425 (35%) were males. Except family history of CAD, all variables were significantly associated to the presence of obstructive CAD. Among 2415 individuals with 1 RF, 654 (27%) had obstructive CAD and these individuals were older and had a higher proportion of men, angina and CCS > 0.

**Table 1:** Baseline characteristics by RF burden and presence of obstructive CAD

Characteristic	0 RF				1 RF			
	Total	Obstructive CAD^b^		*P* value	Total	Obstructive CAD		*P* value
	*N* = 1201	Yes(*N* = 363)	No(*N* = 838)		*N* = 2415	Yes(*N* = 654)	No(*N* = 1761)	
Age^a^	56.26 ± 10.61	61.24 ± 10.69	54.10 ± 9.83	< 0.0001	57.20 ± 10.56	61.87 ± 10.13	55.47 ± 10.22	< 0.0001
Male	425 (35)	188 (52)	237 (28)	< 0.0001	1233 (51)	400 (61)	833 (47)	< 0.0001
Diabetes	–	–	–	–	269 (11)	76 (12)	193 (11)	0.6962
Hypertension	–	–	–	–	949 (39)	263 (40)	686 (39)	0.5989
Hyperlipidemia	–	–	–	–	563 (23)	140 (21)	423 (24)	0.1974
Smoking	–	–	–	–	634 (26)	174 (27)	460 (26)	0.8450
Family history	212 (18)	63 (17)	149 (18)	0.7379	483 (20)	131 (20)	352 (20)	0.9544
Chest pain				< 0.0001				< 0.0001
Nonanginal	433 (36)	53 (15)	380 (45)		893 (40)	92 (14)	801 (45)	
Atypical anginal	599 (46)	165 (45)	394 (47)		1048 (43)	314 (48)	734 (42)	
Typical anginal	209 (18)	145 (40)	64 (8)		474 (20)	247 (38)	227 (13)	
CCS				< 0.0001				< 0.0001
0	548 (46)	64 (18)	484 (58)		1134 (47)	94 (14)	1040 (59)	
0–100	344 (29)	115 (32)	229 (27)		607 (25)	197 (30)	410 (23)	
100–400	231 (19)	115 (31)	116 (14)		431 (18)	180 (28)	251 (14)	
> 400	78 (6)	69 (19)	9 (1)		243 (10)	182 (28)	61 (4)	

Values are presented as *n* (%) unless stated otherwise

*CAD* coronary artery disease, *CCTA* coronary computed tomographic angiography, *CCS* coronary calcium score, *RF* risk factor

^a^Years, mean ± standard deviation

^b^Obstructive CAD was defined as present if an individuals had at least one lesion with ≥50% diameter stenosis or any non-assessable segments due to severe calcification on CCTA

Comparison of discrimination using AUC and IDI is shown in Table 2. The AUC for GEM was significantly larger than that for CONFIRM score, no matter in individuals with 0 (0.843 v.s. 0.762, *p* < 0.0001) or 1 (0.823 v.s. 0.752, *p* < 0.0001) RF. Compared to CONFIRM score, GEM demonstrated a positive IDI in individuals with 0 RF (5%, *p* < 0.0001) and individuals with 1 RF (8%, *p* < 0.0001), respectively.

**Table 2:** Discriminations of CONFIRM score and GEM in individuals with 0 and 1 RF

	AUC			IDI			
	Statistic	95% CI	*P* value	PTP		Statistic^b^	*P* value
				Positive patients^a^	Negative patients		
0 RF							
CONFIRM score	0.756	0.731 to 0.781	< 0.0001	44%	18%	5%	< 0.0001
GEM	0.843	0.820 to 0.866		46%	15%		
1 RF							
CONFIRM score	0.762	0.742 to 0.783	< 0.0001	48%	22%	8%	< 0.0001
GEM	0.823	0.804 to 0.841		55%	21%		

*AUC* area under the receiver operating characteristic curve, *IDI* integrated discrimination improvement, *CI* confidence interval, other abbreviations as in Table 1

^a^Obstructive CAD was defined as present if an individuals had at least one lesion with ≥50% diameter stenosis or any non-assessable segments due to severe calcification on CCTA

^b^Compared to CONFIRM score, the IDI of GEM = [P(GEM|Positive)- P(GEM|Positive)]-[P(CONFIRM score|Negative)- P(CONFIRM score|Negative)]

During a median follow-up of 17 months (interquartile range, 9–23 months), 137 (3.8%) individuals were lost on follow-up. MACEs occurred in 126 individuals (3.5%), including 4 (0.1%) cardiovascular deaths, 11 (0.3%) nonfatal MIs, 46 (1.3%) unstable angina, and 65 (1.8%) late revascularizations. In individuals with 0 RF, GEM had a significantly better discriminatory ability for MACEs than CONFIRM score (AUC for GEM: 0.785 v.s. AUC for CONFIRM score: 0.703, *p* < 0.0001). Results were similar among individuals with 1 RF (AUC for GEM: 0.802 v.s. AUC for CONFIRM score: 0.709, *p* < 0.0001).

Table 3 shows the classification of individuals with 0 RF. Of the 838 negative individuals, by GEM, 269 were correctly reclassified to a lower category, but 72 to a higher category. Of the 363 positive individuals, 64 were correctly reclassified to a higher category but 41 to a lower category. Thus, compared to CONFIRM score, the NRI for GEM was 23.51% in negative, 6.43% in positive, and 29.85% overall (*p* < 0.0001). Results were similar among individuals with 1 RF (Table 4). The NRI for GEM compared to was as follow: 19.42% for negative, 1.68% for positive and 21.10% overall (*p* < 0.0001).

**Table 3:** Reclassification table using PTP categories < 15%, 15–85%, and > 85% (Individuals with 0 RF)

PTP category based on GEM	PTP category based on CONFIRM score				Reclassification^a^		NRI^b^	*p* value for NRI
	Low	Medium	High	Total	Up	Down		
Negative individuals					8.59%	32.10%	29.85%	< 0.0001
Low	300	260	3	563				
Medium	66	196	6	268				
High	3	3	1	7				
Total	369	459	10	838				
Positive individuals^c^					17.63%	11.29%		
Low	11	15	5	31				
Medium	6	235	21	262				
High	1	57	12	70				
Total	18	307	38	363				

*NRI* net reclassification improvement; other abbreviations as in Table 1

^a^Individuals was reclassified by GEM and was compared to CONFIRM score

^b^NRI = [P(Up|Positive)- P(Down|Positive)]-[P(Up|Negative)- P(Down|Negative)]

^c^Positive individuals was defined as those had at least one lesion with ≥50% diameter stenosis or any non-assessable segments due to severe calcification on CCTA

**Table 4:** Reclassification table using PTP categories < 15%, 15–85%, and > 85% (Individuals with 1 RF)

PTP category based on GEM	PTP category based on CONFIRM score			Total	Reclassification^a^		NRI^b^	*P* value for NRI
	Low	Medium	High		Up	Down		
Negative individuals					16.64%	36.06%	21.1%	< 0.0001
Low	418	592	11	1021				
Medium	252	410	32	694				
High	15	26	5	46				
Total	685	1028	48	1761				
Positive individuals^c^					13.15%	11.47%		
Low	19	46	5	70				
Medium	11	439	24	474				
High	3	72	35	110				
Total	33	557	64	654				

Abbreviations as in Table 3

^a^Individuals was reclassified by GEM and was compared to CONFIRM score

^b^NRI = [P(Up|Positive)- P(Down|Positive)]-[P(Up|Negative)- P(Down|Negative)]

^c^Positive individuals was defined as those had at least one lesion with ≥50% diameter stenosis or any non-assessable segments due to severe calcification on CCTA

CONFIRM score classified 55% (459/838) negative individuals with 0 RF and 58% (1028/1721) negative individuals with 1 RF into medium PTP group, for which noninvasive testing were recommend according to current guidelines. Using GEM instead of CONFIRM score would imply a change for diagnostic strategy in these individuals: 57% (260/459) with 0 RF and 58% (592/1028) with 1 RF into low PTP group, for which no further test was recommend. What’s more, among the 852 individuals, only 8 MACEs occurred (0.9%, no cardiovascular death, 1 nonfatal MI, 3 unstable angina, and 4 late revascularizations).

**Fig. 1:**
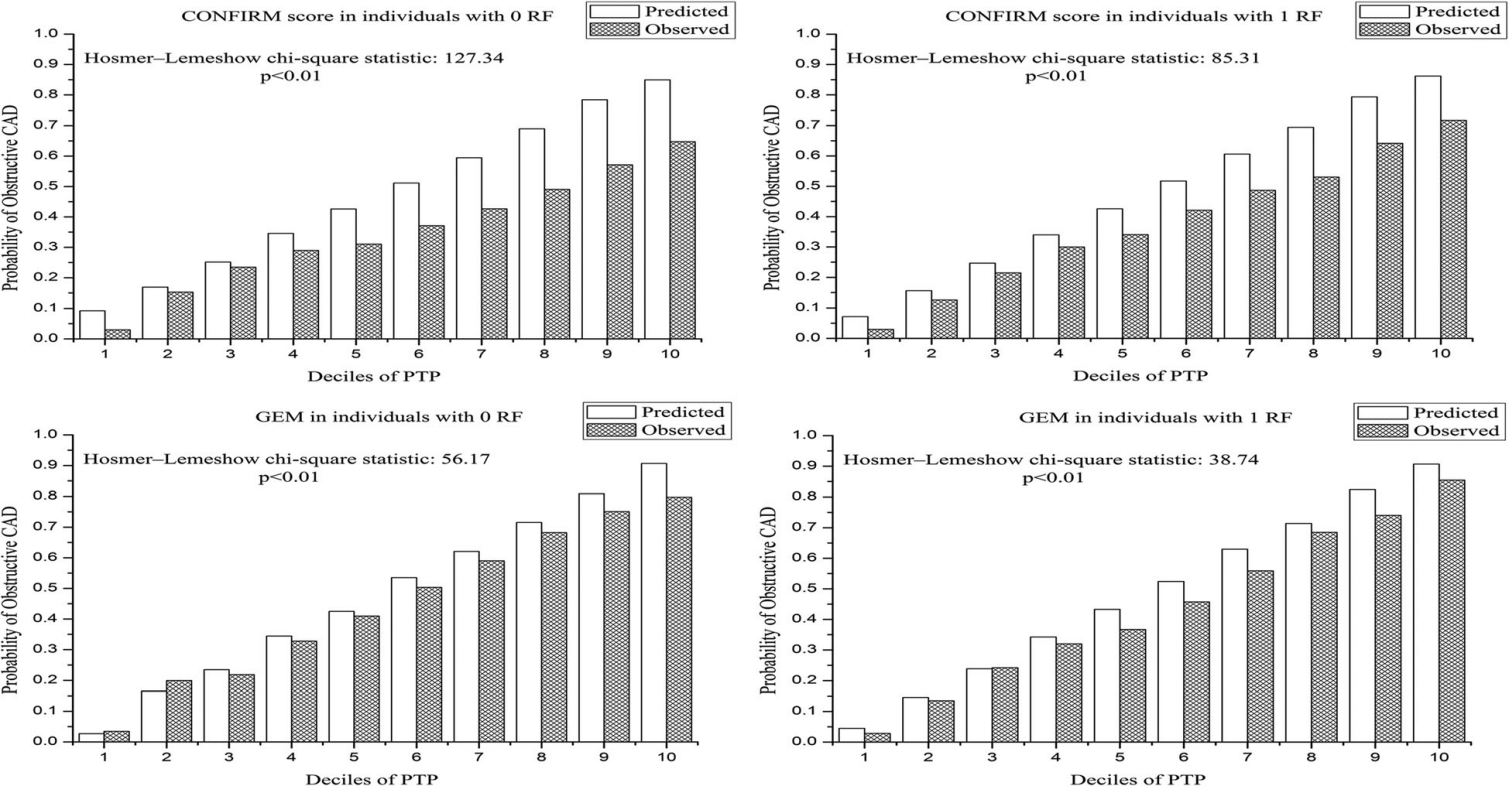
Predicted and observed probabilities of obstructive CAD by deciles of PTP. CAD = coronary artery disease; RF = risk factor; PTP = pretest probability; GEM = Genders extended model

Comparisons of predicted and observed probabilities of obstructive CAD were made by deciles of PTP in Fig. 1. In individuals with 0 RF, CONFIRM score overestimated the prevalence of obstructive CAD resulting in a poor calibration (H-L χ^2^ = 127.34, *p* < 0.01). On the contrary, GEM revealed a lower but still significant degree of discordance between observed and predicted probabilities (H-L χ^2^ = 56.17, *p* < 0.01). Comparably, in individuals with 1 RF, GEM was more well calibrated, whereas calibration for both models was unsatisfactory (CONFIRM score: H-L χ^2^ = 85.31, *p* < 0.01, GEM: H-L χ^2^ = 38.74, *p* < 0.01).

## Discussion

This CCTA-based study completed in individuals at low extreme of traditional CAD RF burden (0 or 1 RF) demonstrated that the addition of CCS in GEM provided a more accurate estimation for PTP of obstructive CAD. Compared to CONFIRM score, GEM showed a larger AUC, a positive NRI and less discrepancy between observed and predicted probabilities in individuals with 0 and 1 RF, respectively. What’s more, using GEM instead of CONFIRM score could change diagnostic strategy in these individuals, resulting a decrease in unnecessary testing.

Although ESC guidelines recommend UDFM as the model to estimate PTP of obstructive CAD, it revealed significantly overestimates in several external validation studies completed in CCTA-based cohorts [7–9]. To address this shortcoming, the medical history-based CONFIRM score was developed from an international cohort of patients undergoing CCTA [10]. The CONFIRM score underwent external validation only once after its publication, showing a positive NRI and less miscalibration, but a similar AUC compared to UDFM. In consideration of the change in the quantitative relationship between CAD and variables in traditional age, sex, chest pain typicality and RF-based approaches [8, 20–22], many efforts have been made to explore whether newer markers could improve the precision of PTP models. A recent work has emphasized that the incorporation of CCS into Duke clinical score improved the diagnostic accuracy for obstructive CAD compared with Duke clinical score alone [23]. Two external validation study [7, 8] for GEM also demonstrated that the addition of CCS promoted the estimation of PTP in the ability of discrimination, classification and calibration, which was confirmed by the present study in individuals with 0 or 1 RF. What’s more, despite the sub-optimal calibration for both models possibly caused by ethnic variation, our results suggested that GEM including CCS provided a more accurate prediction of obstructive CAD than CONFIRM score in individuals at low extreme of traditional RF burden.

So far as we know, this is the first study that systematically validates and compares PTP models in individuals at low extreme of traditional RF burden. In the contemporary environment of rising healthcare costs, a better strategy to select individuals who might benefit from further testing is needed in daily clinical practice [24]. However, several potential reasons may account for the difficult decision-making of diagnostic strategy for symptomatic individuals at low extreme of traditional RF burden, such as lack of awareness, pursuit of economic benefits and fears about the increase in malpractice liability [25, 26]. Current guidelines recommended noninvasive testing, e.g. CCTA and treadmill exercise testing as the appropriate diagnostic test for individuals with medium PTP [4, 5]. Unfortunately, several large and real-world trials which were completed in symptomatic individuals with low-to- medium risk revealed low rates of cardiovascular event and positive noninvasive testing [22, 27–29]. In conformity with this, according to the reclassification table in our study, CONFIRM score classified more than half of the negative individuals into medium PTP group, which may cause overuse of noninvasive testing. Conversely, GEM classified most negative individuals into low PTP group, resulting in a positive NRI and IDI. Although IDI of GEM over CONFIRM was modest, even small improvements can become significant when applied to the large number of low risk individuals evaluated for suspected CAD in everyday practice. What’s more, the rate of MACEs in individuals reclassified into low PTP group was extremely low. Thus, the addition of CCS into PTP model could change the diagnostic strategy safely and effectively, leading to an evident decrease of unnecessary testing.

There were several limitations that warrant acknowledgement. First, this cohort is a subset of a retrospective and single-center study. In real clinical practice, a substantial proportion of patients with stable chest pain are directly referred for other testing based on individual physician decision. Second, CCTA oftentimes overestimates the severity of calcified plaques because of the high-density artifacts [30]. We defined unassessable segments due to severe calcification as positive, so that if these segments were assessable and taken into account, any overestimation would increase further. Thus, this hypothesis would not qualitatively change the conclusions in this analysis. Last, in the future, the conclusions of this study need to be validated and confirmed in comparative cost-effectiveness analyses with long-term outcome data.

## Conclusions

In individuals with 0 or 1 RF, the addition of CCS in GEM provided a more accurate estimation for PTP of obstructive CAD, due to the improvement in discrimination, classification and calibration compared to CONFIRM score. The application of GEM instead of CONFIRM score could change the diagnostic strategy and avoid unnecessary noninvasive testing in individuals at low extreme of traditional RF burden of CAD.

## Abbreviations

AUC: Area under receiver-operator characteristic curve
CAD: Coronary artery disease
CCS: Coronary calcium score
CCTA: Coronary computed tomographic angiography
ESC: European Society of Cardiology
GEM: Genders extended model
H-L: Hosmer–Lemeshow
IDI: Integrated discrimination improvement
MACE: Major adverse cardiovascular event
MI: Myocardial infarction
NRI: Net reclassification improvement
PTP: Pretest probability
RF: Risk factor
UDFM: Updated Diamond-Forrester method
